# Expression proteomics of UPF1 knockdown in HeLa cells reveals autoregulation of hnRNP A2/B1 mediated by alternative splicing resulting in nonsense-mediated mRNA decay

**DOI:** 10.1186/1471-2164-11-565

**Published:** 2010-10-14

**Authors:** Nicholas J McGlincy, Lit-Yeen Tan, Nicodeme Paul, Mihaela Zavolan, Kathryn S Lilley, Christopher WJ Smith

**Affiliations:** 1 Department of Biochemistry, University of Cambridge, Tennis Court Road, Cambridge, CB2 1QW, UK; 2 Biozentrum, University of Basel, Klingelbergstr. 50-70, CH 4056 Basel, Switzerland; 3 MRC Laboratory of Molecular Biology, Hills Road, Cambridge, CB2 0QH, UK

## Abstract

**Background:**

In addition to acting as an RNA quality control pathway, nonsense-mediated mRNA decay (NMD) plays roles in regulating normal gene expression. In particular, the extent to which alternative splicing is coupled to NMD and the roles of NMD in regulating uORF containing transcripts have been a matter of debate.

**Results:**

In order to achieve a greater understanding of NMD regulated gene expression we used 2D-DiGE proteomics technology to examine the changes in protein expression induced in HeLa cells by UPF1 knockdown. QPCR based validation of the corresponding mRNAs, in response to both UPF1 knockdown and cycloheximide treatment, identified 17 *bona fide *NMD targets. Most of these were associated with bioinformatically predicted NMD activating features, predominantly upstream open reading frames (uORFs). Strikingly, however, the majority of transcripts up-regulated by UPF1 knockdown were either insensitive to, or even down-regulated by, cycloheximide treatment. Furthermore, the mRNA abundance of several down-regulated proteins failed to change upon UPF1 knockdown, indicating that UPF1's role in regulating mRNA and protein abundance is more complex than previously appreciated. Among the *bona fide *NMD targets, we identified a highly conserved AS-NMD event within the 3' UTR of the *HNRNPA2B1 *gene. Overexpression of GFP tagged hnRNP A2 resulted in a decrease in endogenous hnRNP A2 and B1 mRNA with a concurrent increase in the NMD sensitive isoforms.

**Conclusions:**

Despite the large number of changes in protein expression upon UPF1 knockdown, a relatively small fraction of them can be directly attributed to the action of NMD on the corresponding mRNA. From amongst these we have identified a conserved AS-NMD event within *HNRNPA2B1 *that appears to mediate autoregulation of *HNRNPA2B1 *expression levels.

## Background

Nonsense-mediated mRNA decay (NMD) is one of a number of RNA surveillance pathways that help to ensure the fidelity of gene expression by degrading mRNAs that lack the proper arrangement of translational signals (reviewed in [1-4]). As the name suggests, NMD is responsible for recognizing and degrading mRNAs that contain premature termination codons (PTCs). In mammals, a termination codon is generally defined as premature by its spatial relationship to exon-exon junctions. The presence of one or more junctions at a distance of > 50-55 nucleotides downstream of the termination codon marks the mRNA for destruction [2,4]. The biochemical basis of this effect is an interaction between the exon junction complex (EJC), a large multi-protein complex that is deposited on the mRNA as a result of splicing, and the complex formed at the stop codon by the terminating ribosome during the first round of translation [1,2,4]. This interaction is mediated by the essential NMD factors UPF1, UPF2 and UPF3 (*UP-Frameshift suppressor*, from their original identification in *Saccharomyces cerevisiae *[5]). Furthermore, in a number of metazoans, the phosphorylation state of UPF1 is regulated by the factors SMG1 and SMG5-7 (*Supressor with Morphological defects on the Genitalia*, from their originally identification in *Caenorhabditis elegans *[6]), which is required for NMD to take place [7]. Until recently the position of any downstream EJCs was thought to be the primary determinant of a PTC in mammals. Recent studies, however, have shown that the distance from the PTC to various cues within the 3' UTR (particularly the cytoplasmic poly-A binding protein PABP) can also play an important role in defining termination events as aberrant [3,8-13]. This is in a similar fashion to those organisms, such as *S. cerevisiae*, *C. elegans *and *Drosophila melanogaster*, where the EJC is absent or plays no role in NMD, and is thought to reflect a primordial mechanism of PTC recognition upon which, in mammals, the EJC has been superimposed [3,4,13].

Apart from its role in dealing with unintended errors in gene expression, NMD has a well documented role in regulating the abundance of many physiological transcripts in all model organism examined to date [14-21]. Moreover, many NMD factors are now known to have additional functions extending beyond NMD (reviewed in [4]). The difference between species in those genes regulated by NMD is thought to be the cause of the differing phenotypes of animals in which Upf1 has been removed [16,19,22]. Amongst these, *Mus musculus *lacking *Upf1 *are embryonic lethal [23], indicating that Upf1, and presumably NMD, plays an important role in mammalian physiology and development. NMD-regulated transcripts can be divided into two broad categories. First those mRNAs that "normally" possess a PTC. These include transcripts that contain upstream open reading frames (uORF) within their 5' UTR, or in which a PTC is introduced as the result of a regulated alternative splicing event (AS-NMD), including those transcripts with an intron more than 50-55 nt into the 3'UTR such that the coding sequence (CDS) termination codon appears premature [15,16,24-26]. Secondly, those mRNAs where NMD is co-opted as a decay mechanism through the interaction of UPF1 with a protein that recognizes a specific set of mRNAs. Two examples are staufen-1 (STAU1) mediated decay (SMD) and the decay of certain replication-dependent histone mRNAs at the end of S-phase of the mammalian cell cycle [27-29]. In both cases a protein recognizes a specific *cis*-element within the 3' UTR and also interacts with UPF1 [27-29]. mRNA degradation is then triggered in a fashion that is dependent on UPF1 and active translation, but independent of the other UPF proteins [27-29].

Previous large-scale investigations into the role of UPF1/NMD in regulating physiological gene expression in metazoans have focused on changes in mRNA abundance [15,20,21,29-34]. In this study we have sought to deepen our understanding of the role of UPF1 in regulating physiological gene expression by examining the changes in protein expression in response to siRNA mediated depletion of UPF1 in HeLa cells, using the expression proteomics technique 2D difference gel electrophoresis (2D-DiGE). We observed a large number of alterations in protein levels; both increases and decreases. By analyzing the levels of the corresponding mRNAs after treatment with either siRNAs against UPF1 or the translation inhibitor cycloheximide we were able to identify a small group of *bona fide *NMD targets; indicating that UPF1's role in regulated gene expression may be more limited than previously thought. However, the majority of these *bona fide *NMD targets contained recognizable NMD-activating features, such as splicing dependent PTCs, introns in the 3'UTR and uORFs. From among these we identified a highly conserved AS-NMD event with the 3' UTR of the *HNRNPA2B1 *gene, which appears to be involved in the autoregulation of *HNRNPA2B1 *mRNA levels.

## Results

### mRNA stabilized as a result of the inhibition of NMD is translated to yield protein

**Figure 1:**
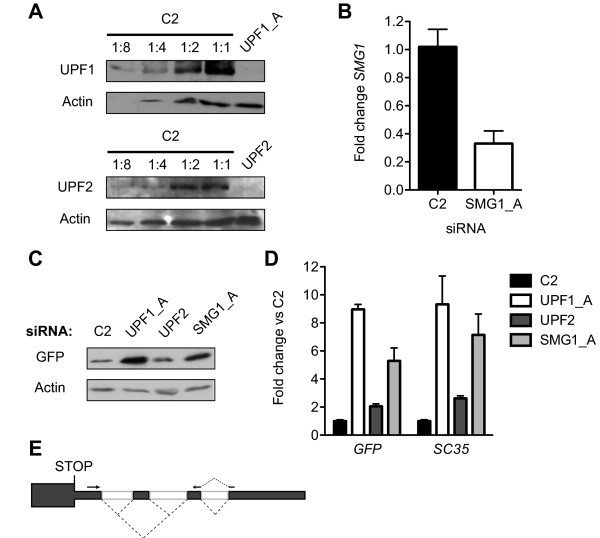
**Knockdown of UPF1, UPF2 and SMG1 results in the production of GFP protein by cells stably expressing pGFPint**. **A** Upper panel: western blot of 10μg total cell protein for UPF1 and actin as a loading control. Lower panel: western blot of 30 μg total cell protein for UPF2 and actin as a loading control. In each case the C2 treated sample was diluted 1:2, 1:4 and 1:8 in RIPA buffer in order to better estimate the degree of knockdown in the knockdown sample. Representative samples of three biological repeats are shown. **B**. Histogram comparing the fold change in SMG1 mRNA levels in response to treatment with C2 (black bar) or SMG1_A (light grey bar) siRNAs, as measured by QPCR on parallel RNA samples. The height of each bar represents the mean of three biological repeats, while error bars represent the standard error of the mean (SEM). **C**. Western blot of 10 μg total cell protein for GFP and actin as a loading control. Representative samples of three biological repeats for each siRNA are shown. **D**. Histogram comparing the fold change in mRNA levels for GFP and the NMD sensitive isoforms of SC35 (1.6 kb and 1.7 kb, [36]) in response to treatment with each siRNA, as measured by QPCR on parallel RNA samples. The height of each bar represents the mean of three biological repeats, while error bars represent SEM. The colour scheme is indicated in the side panel. **E**. Schematic of AS-NMD events within the 3' UTR of *SC35 *(*SFRS2*). Dark boxes represent exons, and white boxes introns. Dashed lines denote alternative splicing patterns and arrows denote the QPCR primers used in **D**.

Before embarking upon a proteomic analysis of the consequences of UPF1 knockdown we carried out a proof of principle experiment to demonstrate that mRNAs stabilised by inhibition of NMD could be translated to yield protein. To this end we constructed the pGFPint reporter plasmid, containing an efficiently spliced artificial intron (based on α-tropomyosin exons 2 and 3 and a 111nt intron from β-globin [35]) in the 3' UTR. Splicing of the intron creates an exon-exon junction 105 nucleotides downstream of the GFP stop codon, making it appear premature and hence the mRNA NMD sensitive. A cell line stably expressing pGFPint was constructed and subjected to knockdown of NMD factors UPF1, UPF2 and SMG1 or transfection with a control siRNA, C2. Depletion of UPF1 and UPF2 protein was achieved to levels less than 12.5% of control, as estimated by western blot (Figure 1A). In the absence of an antibody against SMG1 the reduction in *SMG1 *mRNA was measured by QPCR. A reduction to levels approximately 35% of control was achieved (Figure 1B). Both GFP mRNA (Figure 1D, left) and protein (Figure 1C) showed a large increase in response to UPF1 knockdown, a much smaller increase in response to UPF2 knockdown and an intermediate response to SMG1 knockdown. Analysis of pGFPint mRNA by RT-PCR indicated that the artificial intron was entirely spliced (data not shown).

To further examine the differing response of pGFPint to the knockdown of different NMD factors, the levels of the NMD sensitive isoforms of *SC35 *(*SFRS2*) were examined by QPCR (1.6 and 1.7 kb [36], Figure 1D, right. Schematics of events in Figure 1E). Interestingly, the pattern of changes in the *SC35 *isoforms mirrored that of pGFPint mRNA: UPF1 knockdown provoked the largest fold change followed by SMG1 and then UPF2. Given the high degree of knockdown achieved for all the factors examined, it appears that NMD of pGFPint and *SC35 *1.6 and 1.7 kb mRNA is differentially sensitive to the knockdown of UPF1, UPF2 or SMG1. This phenomenon has been described previously and is thought to reflect distinct branches of the NMD pathway with differential requirements for UPF2 [37]. Since the inception of this work two further reports of similar NMD reporter systems support our conclusion that mRNAs stabilized as a result of NMD inhibition are active substrates for translation [38,39]. Indeed, this idea is also borne out by published data from more physiological circumstances [40-42].

### A multi-gel 2D-DiGE study of UPF1 knockdown in HeLa cells reveals numerous changes in protein expression

To identify global changes in protein expression in response to UPF1 knockdown we performed a multi-gel 2D-DiGE study of HeLa cells in which UPF1 had been depleted by RNA interference (RNAi). Samples were harvested 48 h after the second siRNA hit on the basis that this provided sufficient time to observe the primary consequences of UPF1 knockdown, while minimizing any secondary indirect effects. The basic principle of 2D-DiGE is that protein extracts from two different biological situations (in this case HeLa cells treated with a control or targeted siRNA) are differentially labelled with fluorescent dyes before being mixed and fractionated by 2D gel electrophoresis [43,44]. The fluorescent image of the gel is then examined to identify protein spots where one fluorescent dye predominates indicating the change in expression of one or more of the constituent proteins between the two biological situations [43,44]. The protein spot is then excised from the gel and its composition determined by mass spectrometry [43,44].

**Figure 2:**
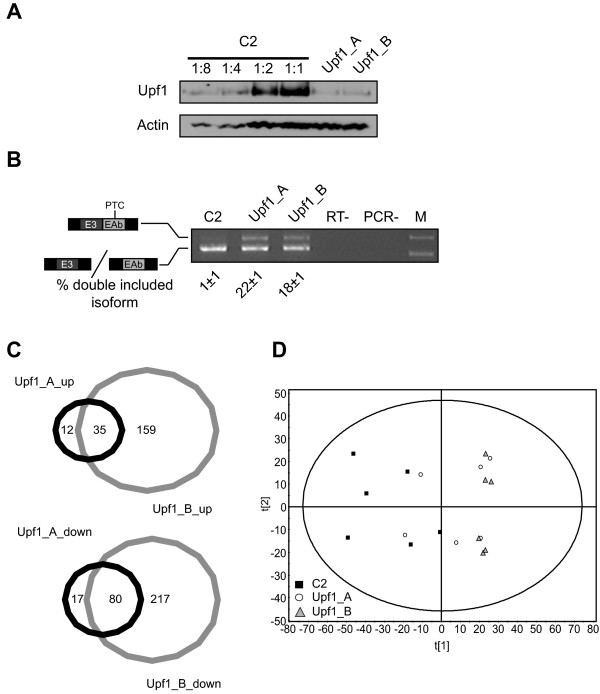
**Changes in protein spot expression in response to UPF1 knockdown**. **A**. Representative western blot of 10 μg total cell protein for UPF1 and actin as a loading control for each sample. C2 treated sample was diluted 1:2, 1:4 and 1:8 in ASB14 buffer in order to estimate the degree of knockdown. **B**. Representative U2AF^35 ^RT-PCR assay on parallel RNA samples. The adjoining cartoon illustrates the identity of each band. The lower band results from alternative inclusion of one of a pair of normally mutually exclusive exons of equal size, termed E3 (yielding isoform U2AF^35^a) and EAb (yielding isoform U2AF^35^b) [45]. The upper band represents inclusion of both exons, which results in a frameshift that creates a PTC - making isoform U2AF^35^c NMD sensitive [45]. M: 1 kb plus marker (GE healthcare). RT-: addition of RT performed without reverse transcriptase. PCR-: PCR performed without template. Underlying numbers indicate the percentage of the signal from both bands represented by he upper, double-included NMD-sensitive, band for each siRNA treatment (mean of 6 biological replicates ± SEM). **C**. Proportional Venn diagrams representing the number of protein spot changes unique and common to each siRNA against UPF1. The upper, red coloured, diagram details upward changes whereas the lower, blue coloured diagram details downward changes. **B**. Principal component analysis (PCA) scores plot illustrating the similarity of the multi-gel study samples to each other by their relationships with the first two principal components (PCs) describing the whole multi-gel study dataset. Blue squares - C2 treated samples; red circles - Upf1_A treated samples, green triangles - Upf1_B treated samples. t[1] - score relating to PC_1_; t[2] - score relating to PC_2_.

In order to identify changes that were specific to UPF1 knockdown, we examined the response of HeLa cells to treatment with two different siRNAs against UPF1, Upf1_A and Upf1_B, compared to a control siRNA, C2 (N = 6 for each condition). The efficacy of UPF1 knockdown, as assessed by western blot, was > 75% for both siRNAs (Figure 2A). Effective depletion of UPF1 was further confirmed in parallel RNA samples by RT-PCR for an AS-NMD event within U2AF^35 ^[45] (Figure 2B). The proportion of the NMD sensitive upper isoform, U2AF^35^c, was clearly enriched upon treatment with Upf1_A or Upf1_B, confirming functional impairment of the NMD pathway.

The 2D-DiGE multi-gel study resulted in identification of a large number of both upward and downward changes in protein expression (p < 0.01, Student's t-test, two tails). As expected, many but not all changes were common to both siRNAs (Figure 2C). Of the 3081 spots detected in the analysis, 47 spots increased in expression in response to Upf1_A treatment while 194 spots increased in expression in response to Upf1_B treatment. Of the upward changes, 35 were common to both siRNAs. Furthermore, Upf1_A treatment resulted in 97 spots decreasing in abundance while 297 spots decreased in abundance in response to Upf1_B treatment. Of the downward changes, 80 were common to both siRNAs.

Multivariate statistical analysis provides an alternative method to Student's t-test for identifying patterns in large multivariate datasets such as that generated by the 2D-DiGE multi-gel study [46]. Abundance data from all spots (3081) for all samples was subject to principle component analysis (PCA) to examine how the experimental samples clustered on the basis of all spot changes. The scores scatter plot for the first two principal components revealed a separation between C2 and the two UPF1 knockdown conditions along PC_1 _(Figure 2D). Interestingly, there is no apparent separation between Upf1_A and Upf1_B treated samples. This was also the case when other PCs were examined (data not shown). This suggests that despite the appearance that some spots change with one siRNA but not the other, most spots undergo correlated changes in response to both siRNAs, although one change or the other may not achieve significance in the univariate sense.

In order to identify which proteins were responsible for the observed increases in expression, and hence are candidate UPF1 targets, 85 of the protein spots that had increased in expression were excised for identification by mass spectrometry (Additional file 1 1). Spots were picked first from those that had changed significantly in response to both Upf1_A and Upf1_B. Then, on the basis of the PCA, others were picked that had changed significantly in response to either siRNA, starting with those that had narrowly escaped significance with the second siRNA. 17 down-regulated spots were also picked, from those that had decreased significantly in response to both siRNAs (Additional file 1). Of the 85 up-regulated spots, 58 yielded sufficient material to allow protein identification, whereas of the 17 down-regulated spots, 13 yielded sufficient material. For each spot, potential protein constituents were identified from liquid chromatography-tandem mass spectrometry of trypic peptides produced by in-gel digestion, using the MASCOT search engine [47]. As a result, 128 unique proteins were identified from up-regulated spots and 21 from down-regulated spots. Some proteins were identified in more than one spot, including 6 that were found in both up- and down-regulated spots. This could be due to post-translational modification of the proteins, or the existence of isoforms that are different in size or pI, but are indistinguishable by their tryptic peptide pattern. Information on the identified proteins and peptide sequences, along with the change observed for each spot is detailed in Additional file 2.

### Validation of NMD targeted mRNAs

In order to determine which of the identified proteins represented *bona fide *NMD targets, two rounds of QPCR validation were employed. In the first round we tested whether levels of the mRNAs corresponding to the identified proteins were affected by UPF1 knockdown mediated by Upf1_A siRNA. Since NMD and the other UPF1-dependent mRNA decay pathways (SMD and histone mRNA decay) are dependent on active translation [27,28,48], we next measured changes in mRNA levels following treatment of cells with the translation inhibitor cycloheximide. For each gene of interest QPCR primers were designed to mRNA regions not known to undergo alternative splicing, and eight replicate samples were used in order to achieve the same statistical power as the 2D-DiGE multi-gel study [49,50]. Only genes that showed statistically significant increases (p < 0.05, Student's t-test, one-tail) in response to both treatments were deemed to be genuine targets of NMD/UPF1-dependent mRNA decay.

**Figure 3:**
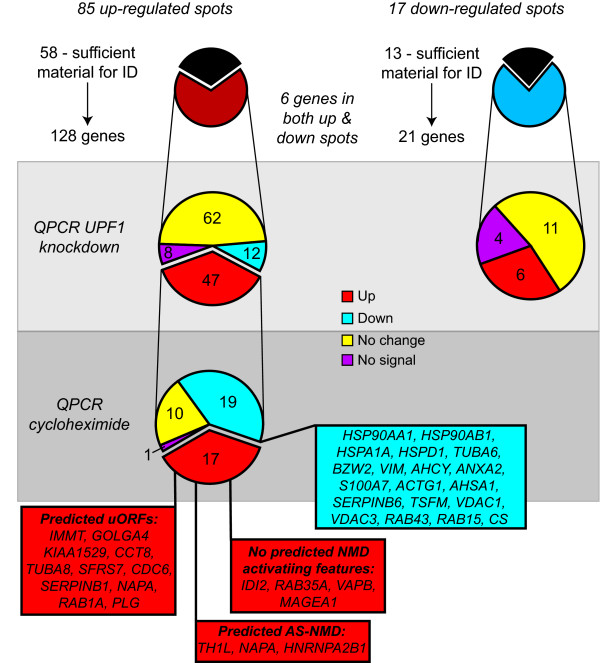
**Summary of validation results**.

The results of the QPCR validation are detailed in Additional file 2 and summarised in Figure 3. Of the 128 mRNAs corresponding to proteins from up-regulated spots, 47 (37%) increased in response to UPF1 knockdown, as expected of UPF1/NMD targets. A further 62 (48%) showed no significant change in expression, 12 decreased (9%), while 8 (6%) failed to produce an intelligible signal. Strikingly, from the 17 down-regulated protein spots none of the 21 corresponding mRNAs was down-regulated upon UPF1 knockdown. Eleven did not change significantly in expression, 4 failed to show an intelligible signal, while the remaining 6 actually increased in expression (Additional file 2). While 3 of these 6 proteins were among those also found in up-regulated spots, it is striking that not a single one of the genes corresponding to the protein constituents of down-regulated spots showed an accompanying decrease in mRNA expression. This suggests that UPF1 knockdown has a negative role in the translational efficiency of at least some of these proteins.

The 47 validated UPF1 target genes from up-regulated spots were then subjected to the second round of validation by cycloheximide treatment. Of the 47 UPF1 targets, 17 (34%) increased in expression, as expected of authentic NMD targets (*IMMT, GOLGA4, IDI2, TH1L, PLG, KIAA1529, CCT8, TUBA8, SFRS7, MAGEA1, CDC6, SERPINB1, NAPA, HNRNPA2B1, VAPB, RAB1A, RAB35*). Of the remainder, one (*CANX*) failed to give an intelligible signal, 10 (21%) showed no significant change in expression, while 19 (40%) actually decreased in expression. The 30 genes that did not increase in expression in response to cycloheximide treatment may represent secondary effects of UPF1 knockdown or targets of UPF1 in processes other than NMD.

With reference to the original 2D DiGE analysis, of the 58 upregulated spots that yielded protein identifications, 34 (59%) contained at least one protein whose corresponding mRNA was validated as being upregulated by knockdown of UPF1, and in 26 cases (45%) the upregulated mRNA corresponded to the major protein component of the spot, as indicated by MASCOT scores (see Additional file 2). Of the 34 spots, 18 (i.e. 31% of the total) contained proteins that were also upregulated by cycloheximide treatment. In five cases (*CCT8, SERPINB1, NAPA, HNRNPA2B1, RAB1A*) the doubly validated NMD target corresponded to the major protein constituent of the protein spot, as judged by MASCOT scores, and so provides a clear explanation for the observed upregulation. A number of the other NMD targets were among the more abundant components of the spots in which they were identified (e.g. *IMMT, VAPB, TUBA8, RAB35*). In the remaining cases (e.g. *GOLGA4, IDI2, TH1L, PLG, SFRS7, MAGEA1, CDC6, KIAA1529*), the doubly validated NMD targets represented relatively minor constituents of their resident spots. In four of these cases the spot also contained a more abundant validated UPF1 target that was not cycloheximide upregulated.

The remaining 24 spots (41% of the total) contained no protein whose mRNA was upregulated by UPF1 knockdown. This suggests that relatively few of the changes in protein expression upon UPF1 knockdown can be attributed directly to UPF1's role in mRNA decay. This result is in broad agreement with the more extensively validated studies of UPF1's role in physiological gene expression [20,21,34,51]. (discussed below)

### Identification of NMD activating features

We next sought to identify possible NMD activating features within the doubly validated genes. To this end, maximum transcript alignments [52] of corresponding Unigene clusters [53] were generated using SPA [54] and examined for instances of: i) introns more than 50 nt downstream of the termination codon of the largest ORF within a transcript, as an indication of AS-NMD. ii) The presence of ORFs upstream of the largest ORF within a maximum transcript, as an indication of the presence of uORFs within the 5' UTR. Potential NMD sensitive maximum transcripts were then scored according to the number of peptides identified by mass spectrometry that were present within the protein sequence encoded by the largest ORF of the maximum transcript. Only transcripts encoding all of the identified peptides were considered as being potentially responsible for the observed upregulation of protein spots. For some genes, this step eliminated many potential NMD substrates. For example, the *SFRS7 *gene encodes the SR splicing regulatory protein 9G8, which has a well characterized AS-NMD event involving a "poison" cassette exon [55]. In this study however, the 9G8 peptides identified by mass spectrometry are not encoded by the alternatively spliced PTC-containing isoform. In contrast, many of the *SFRS7 *maximum transcripts contain one or two additional short uORFs upstream of the main protein coding ORF.

Of the 17 doubly validated genes, we found evidence of at least one NMD feature in 12 cases. Notably, all five doubly validated genes that constituted the major components of their resident spots (*CCT8, SERPINB1, NAPA, HNRNPA2B1, RAB1A*) had at least one NMD feature that was consistent with the peptide data. The most common features, found in 11 cases, were uORFs. In addition, there were three genes (*TH1L*, *NAPA *and *HNRNPA2B1*) with one or more alternative splicing events at a sufficient distance downstream of the main ORF to activate NMD.

### AS-NMD events in *TH1L *and *NAPA*

**Figure 4:**
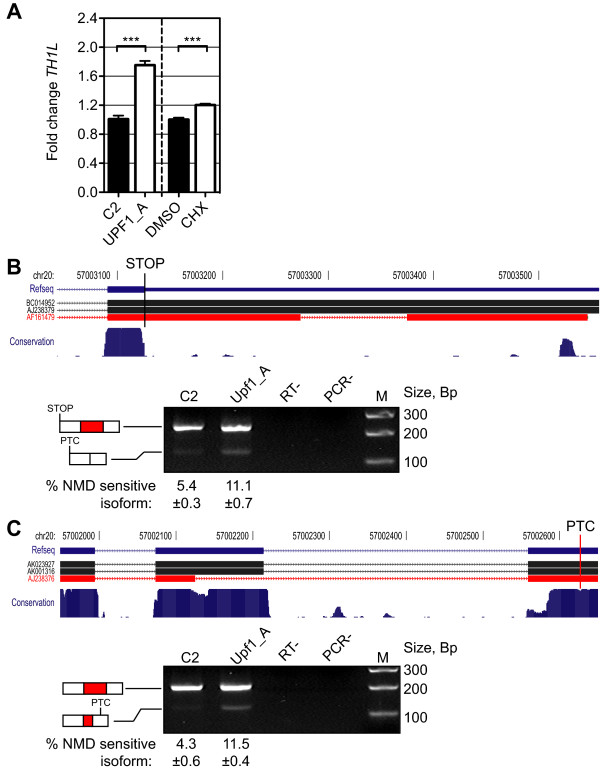
**AS-NMD within the *TH1L *gene**. **A** Histogram representing QPCR validation results for *TH1L*. Bars represent mean fold change in mRNA levels in response to either UPF1 knockdown (left panel, N = 8) or cycloheximide treatment (right panel, N = 8) ± SEM. p-value summary (Student's t test, one tail): * p < 0.05, ** p < 0.01, *** p < 0.001. QPCR primers were located upstream of the schematic shown in **B**, in a region expected to be unaffected by alternative splicing. **B**. Upper panel: schematic of the AS-NMD event within 3' UTR of *TH1L *produced using the UCSC genome browser [114], running 5' left to 3' right. Boxes represent exons while chevroned lines represent introns. The upper blue cartoon indicates the Refseq annotated 3' UTR structure, the thinning of the box indicating the end of the protein coding sequence. The underlying cartoons are Genbank mRNAs illustrating the AS isoforms predicted by our analysis, the red mRNA represents the NMD sensitive isoform - a retained intron is spliced to make the normal stop codon appear premature. The lower blue histogram represents conservation across 17 vertebrate species as calculated by [115]. Lower panel: RT-PCR of the AS-NMD event, illustrating the effect of UPF1 knockdown. M: 1 kb plus marker (GE healthcare). RT-: addition of RT performed without reverse transcriptase. PCR-: PCR performed without template. Underlying numbers indicate the mean (± SEM, N = 3) percentage of the total signal from both bands represented by the NMD-sensitive for each siRNA treatment. **C**. UCSC genome browser schematic and RT-PCR of a second predicted event within *TH1L*. Use of an alternative 5' splice site results in a frameshift creating a downstream PTC. The arrangement of elements is the same as for section **B**.

We next tested each of the AS-NMD predictions by RT-PCR or QPCR. TH1L was a relatively minor constituent of spot 831, which was up-regulated 1.39 fold in response to UPF1 knockdown (Additional file 2). *TH1L *mRNA was up-regulated approximately 1.4 fold in response to UPF1 knockdown and to a similar extent by cycloheximide (Figure 4A). *TH1L *is predicted to contain three AS-NMD events: first, a 101 nt intron within its 3' UTR, which when spliced causes the normal stop codon to appear premature (Figure 4B upper panel). Secondly, retention of the intron between exons 13 and 14. Thirdly, the use of an alternative 5' splice site within exon 13, resulting in a frameshift that creates a PTC (Figure 4C upper panel). The first two events are consistent with the peptide data, while one of the peptides lay downstream of the PTC introduced by use of the internal 5' splice site on exon 13, thus ruling out the latter event as a contributor to the upregulation of protein spot 831. RT-PCR was performed with primers flanking each AS-NMD event to determine whether the PTC containing isoform was stabilised as a result of UPF1 knockdown (Figure 4B and 4C, lower panel). The 3'UTR splicing event and the exon 13 alternative 5' splice site event both showed a significant 2-3 fold increase in the proportion of the NMD sensitive isoform in response to UPF1 knockdown, to a level of approximately 11% (Figure 4B and 4C, lower panel). In contrast, no effect of UPF1 knockdown was seen upon the intron 13 retention event (data not shown). Taken together, the peptide and RT-PCR data therefore suggest that the 3'UTR intron is the NMD feature responsible for the observed upregulation of *TH1L *protein, but that both events contributed to upregulation of its mRNA upon UPF1 knockdown.

**Figure 5:**
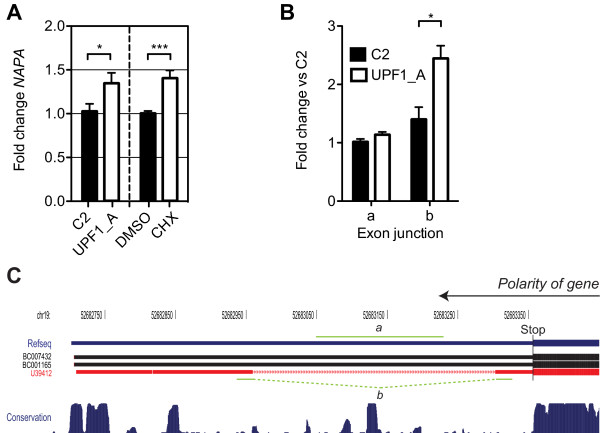
**AS-NMD within the *NAPA *gene**. **A**. Histogram representing QPCR validation results for *NAPA*. Bars represent mean fold change in mRNA levels in response to either UPF1 knockdown (left panel, N = 8) or cycloheximide treatment (right panel, N = 8) ± SEM. p-value summary (Student's t test, one tail): * p < 0.05, ** p < 0.01, *** p < 0.001. QPCR primers were located upstream of the schematic shown in **B**, in a region expected to be unaffected by alternative splicing. **B**. Schematic of the AS-NMD event within 3' UTR of *NAPA *produced using the UCSC genome browser [114]. Boxes represent exons while lines with chevrons represent introns. The upper blue cartoon indicates the Refseq annotated 3' UTR structure, the thinning of the box indicating the end of the protein coding sequence. The underlying cartoons are Genbank mRNAs illustrating the AS isoforms predicted by our analysis, the red mRNA represents the NMD sensitive isoform - a retained intron is spliced to make the normal stop codon appear premature. The lower blue histogram represents conservation across 17 vertebrate species as calculated by [115]. **C**. Histogram representing QPCR validation results of the predicted AS-NMD. Bars represent mean fold change in of the detailed exon junction in response to either UPF1 knockdown (± SEM, N = 3). p-value summary (Student's t test, one tail): * p < 0.05, ** p < 0.01, *** p < 0.001.

NAPA was the major component of spot 1997, which was upregulated ~1.3 fold by UPF1 knockdown (Additional file 2). Likewise, *NAPA *mRNA was upregulated 1.35 - 1.4 fold by UPF1 knockdown and cycloheximide (Figure 5A). *NAPA *has two predicted NMD features that are consistent with the 9 peptides that identified it. An alternatively spliced intron 53 nt into its 3'UTR is at the threshold distance for inducing NMD (Figure 5). In addition, the 5'UTR of a maximum transcript has overlapping 9 and 3 codon uORFs. QPCR was carried out to analyze levels of the isoforms in which the 3'UTR intron was retained or spliced. Levels of *NAPA *mRNA with the intron spliced out (denoted junction *b*) were elevated by ~2.5 fold after UPF1 knockdown, whereas transcripts with the intron retained were not significantly affected (Figure 5B), suggesting that the 3'UTR intron is the feature responsible for the observed upregulation of the NAPA containing spot.

### AS-NMD mediated autoregulation of *HNRNPA2B1*

**Figure 6:**
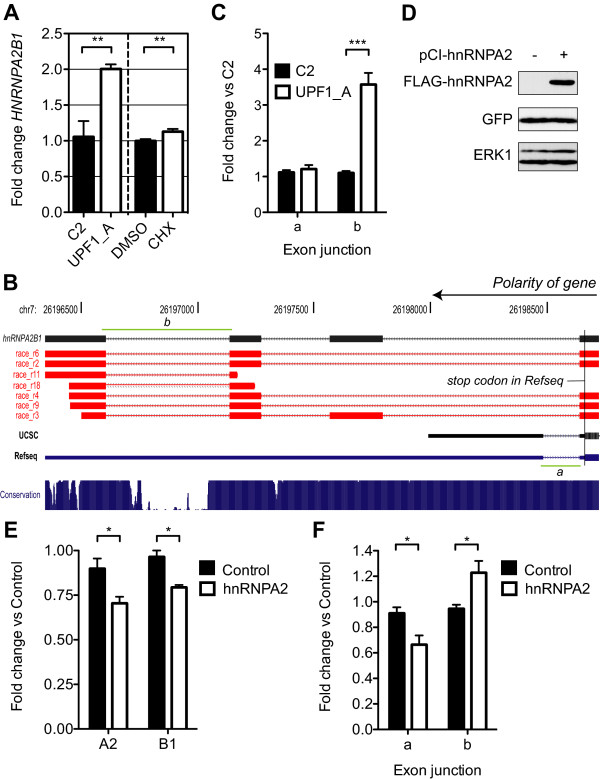
**AS-NMD within the *HNRNPA2B1 *gene**. **A**. Histogram representing QPCR validation results for *HNRNPA2B1*. Bars represent mean fold change in mRNA levels in response to either UPF1 knockdown (left panel, N = 3 in this instance) or cycloheximide treatment (right panel, N = 8) ± SEM. p-value summary (Student's t test, one tail): * p < 0.05, ** p < 0.01, *** p < 0.001. QPCR primers were located upstream of the schematic shown in **B**, in a region expected to be unaffected by alternative splicing. **B**. Schematic of the 3' UTR of *HNRNPA2B1 *produced using the UCSC genome browser [114], running 5' right to 3' left as indicated by the upper arrow. The upper black cartoon represents the prediction of the exonic structure (boxes) predicted by our bioinformatic analysis. The underlying red cartoons represent the highest scoring BLAT alignments [56] of several of the RACE sequences obtained in our analysis. Underlying these are cartoons representing the UCSC prediction of 3' UTR and the Refseq annotated UTR. The lower blue histogram represents conservation across 17 vertebrate species as calculated by [115]. The small UTR intron in the Refseq mRNA is too close to the stop codon for its splicing to make the stop codon appear premature (junction *a*, denoted by the green line). Splicing of the final intron in the UTR (junction *b*, denoted by the green line) would be expected to render all incumbent isoforms NMD sensitve. **C**. Histogram representing QPCR results for exon junctions *a *and *b*. Bars represent mean fold change (N = 3, ± SEM) in mRNA levels in response to UPF1 knockdown. **D**. Western blot of GFP positive HeLa cells sorted by flow cytometry. HeLa cells were co-transfected with a GFP expressing plasmid and either FLAG-tagged hnRNP A2 or an empty vector control. ERK1 was used as a loading control. **E**. Histogram representing QPCR results from parallel RNA samples to **D**. Bars represent mean fold change (N = 3, SEM) of *HNRNPA2 *and *B1 *in response to hnRNPA2 overexpression. p-value summary (Student's t test, two tails): * p < 0.05, ** p < 0.01, *** p < 0.001. **F**. Histogram representing QPCR results from parallel RNA samples to **D**. Bars represent mean fold change (N = 3, SEM) of UTR junctions *a *and *b *in response to hnRNPA2 overexpression. p-value summary (Student's t test, two tails): * p < 0.05, ** p < 0.01, *** p < 0.001.

HNRNPA2B1 was the most abundant protein in spot 2105, which was upregulated 1.3 - 1.7 fold by the two UPF1 siRNAs (Additional file 2). *HNRNPA2B1 *mRNA showed an approximate two-fold increase upon UPF1 knockdown and a small but significant increase upon cycloheximide treatment (Figure 6A and Additional file 2). Bioinformatic analysis indicated that *HNRNPA2B1 *contains extensive alternative splicing within its 3' UTR that would cause the normal stop codon to appear premature (Figure 6B). As an NMD feature this would be consistent with the 5 peptides that identified HNRNPA2B1. The predicted UTR structure is, however, in conflict with the Refseq annotation. In order to confirm the existence of the predicted NMD sensitive isoforms of the 3' UTR, we performed 3' RACE (Rapid Amplification of cDNA Ends) for *HNRNPA2B1 *on mRNA taken from the UPF1 knockdown RNA samples used for validation. The resulting sequences were aligned to the genome using BLAT [56]. The RACE tags clearly support the 3' UTR structure predicted from the unigene cluster; comprising three additional exons after that in which the *HNRNPA2B1 *CDS ends (Figure 6B, Additional file 3). Splicing of the final intron or inclusion of the first additional exon would create an exon-exon junction at sufficient distance downstream to make the normal stop codon appear premature. Splicing of the final intron was examined by exon-junction specific QPCR and found to be up-regulated by 3.5-fold upon UPF1 knockdown (junction *b*, Figure 6B and 6C), whereas a junction in the Refseq mRNA expected not be NMD sensitive (denoted junction *a*, Figure 6B) was not upregulated (Figure 6C). The whole area of the 3' UTR is very highly conserved (Figure 6B), suggesting that AS-NMD might be important in the regulation of *HNRNPA2B1 *levels. Indeed full-length mRNAs and ESTs representing similar UTR structures can also be observed in the orangutan (*Pongo pygmaeus abelii*), other mammals (*M. musculus *and *Bos taurus*) and even the chicken (*Gallus gallus*). While 3' UTRs have many roles in regulating gene expression, any of which may result in the observed conservation [57], many other genes of the hnRNP and SR protein families have been identified as containing such highly conserved AS-NMD events [32,33,55,58-60]. Indeed, murine *Hnrnpa2b1 *was also identified as containing AS-NMD by Ni *et al. *[32], although the event was not the same as reported here (M. Ares, personal communication). Many such conserved AS-NMD events have been show to be subject to autoregulation by their own protein levels [32,36,58,60-66]. We examined whether this is also the case for HNRNPA2B1. FLAG tagged HNRNPA2 isoform or an empty expression vector was transfected into HeLa cells in combination with a GFP expressing plasmid. GFP expressing cells were then isolated by flow cytometry in order to enrich for cells possessing the co-transfected plasmid. Expression of FLAG-HNRNPA2 in the sorted cells was confirmed by western blot (Figure 6D). The expression and splicing of *HNRNPA2B1 *was examined in parallel RNA samples, using primers specific for the endogenous transcripts. Overexpression of FLAG-HNRNPA2 reduced *HNRNPA2 *and *HNRNPB1 *mRNA levels to 75-80% of control (Figure 6E), demonstrating that HNRNPA2 protein can regulate *HNRNPA2B1 *mRNA levels. Examining the effect of overexpression on UTR junctions *a *and *b *indicated that non-NMD sensitive junction *a *was decreased in expression to approximately 60% of control, while NMD sensitive junction *b *was increasing in expression by 20% over control levels (Figure 6F). These results are consistent with HNRNPA2B1 regulating the abundance of its own mRNA by altering splicing of the 3' UTR to promote the production of isoforms degraded by NMD.

## Discussion

### The role of UPF1-dependent mRNA decay in regulating physiological gene expression

We have demonstrated that the knockdown of UPF1 in HeLa cells results in a large number of changes in protein expression but that only a minority of these can be attributed to UPF1's characterized roles in NMD and other translation dependent mRNA decay pathways. This result is in broad agreement with the more extensively validated studies of UPF1's role in physiological gene expression [20,21,34,51], suggesting that NMD plays a more restricted role in regulating gene expression than previously claimed [67,68]. For example, in order to measure the efficiency of NMD in cell lines expressing different amounts of RNPS1, Viegas and colleagues sought to identify genuine NMD target genes from genes identified by microarray as upregulated upon UPF1 knockdown [51]. They examined both the abundance of the fully processed mRNA and pre-mRNA, finding that in the majority of cases (14/16) both were upregulated. This indicates that the increase in mRNA levels was likely to be a transcriptional effect rather than due to the direct action of NMD [51].

The majority of the 17 confirmed NMD targets (defined as UPF1 and translation-dependent) have not been previously identified by microarray studies of UPF1 dependent gene expression in mammals [15,29-32,51,69], indicating the complementarity of proteomic and transcriptomic analyses. However, given the bias of 2D-DiGE towards higher abundance proteins, it may be that this mechanism is more pertinent for genes with lower absolute expression levels. Recent findings have indicated that the distance between the stop codon and poly(A) tail can also be an important determinant of NMD (reviewed in [3]). However, we were unable to find an over-representation of greater 3' UTR lengths within our set of doubly validated genes (data not shown). We believe that it is relevant that all of the identified NMD activating features within the 17 doubly validated NMD targets were either uORFs or introns positioned in the 3'UTR or towards the 3' end of the transcript. As a result the detected proteins would all represent regular full-length isoforms, and would be expected to be stable. The protein products produced from other stabilized AS-NMD substrate mRNAs might be inherently unstable due to their truncated nature. This would argue against the functional roles often proposed for these products [70,71]. In support of this, we observed that the potential truncated PTB isoform, encoded by the AS-NMD targeted mRNA in which exon 11 is skipped [58], is not detectably expressed from cDNA expression vectors (in which there is no NMD activating feature) unless proteasome activity is inhibited by the proteasomal inhibitor MG132 (NJM and CWJS, unpublished observation). This suggests that the inherent instability of some protein products might preclude their identification as NMD targets by proteomic approaches. Nevertheless, a pilot DiGE experiment did not reveal a noticeable increase in upregulated spots when UPF1 knockdown was analyzed under conditions of proteasome inhibition by MG-132 (NJM and CWJS, unpublished observation), suggesting that our current investigation has not suffered substantially from this potentially confounding effect.

In addition to identifying genuine NMD targets, our validation strategy also highlighted a number of other interesting groups. First, those proteins whose mRNA was not up-regulated by UPF1 knockdown may represent targets of nonsense mediated translational repression (NMTR), wherein PTC containing mRNAs apparently escape NMD but do not produce detectable levels of protein [72,73]. Secondly, those genes that were upregulated by UPF1 knockdown but not upregulated by cycloheximide treatment. An interesting feature of these 19 unusual UPF1 targets is an enrichment of heat shock proteins of the hsp90, hsp70 and hsp60 classes (*HSP90AA1, HSP90AB1, HSPA1A, HSPD1, AHSA1*). In *S. cerevisiae *a specialized mRNA decay pathway, termed initiation dependent decay, operates under conditions where translation initiation is down-regulated but not abolished [74,75]. Of particular interest, initiation dependent decay targets a variety of heat shock proteins, including hsp70 and 90, and is dependent upon UPF1 and UPF2. While our experiments were not carried out under conditions where translation initiation is expected to be impaired, they suggest that initiation dependent decay may be relevant to mammalian systems. However, this observation could also be the result of a shared transcriptional activator of these heat-shock proteins being NMD sensitive, but generally translationally down-regulated by cyclohexamide. Thirdly, analysis of the spots that were down-regulated upon UPF1 knockdown indicate yet another potentialiy interesting group of UPF1 targets. None of the mRNAs corresponding to the protein constituents of these spots were down-regulated by UPF1 knockdown; indeed some were actually upregulated. This suggests that UPF1 may play a role in the synthesis or degradation of these proteins. Given UPF1's known roles, it seems more likely that it acts at the translational level and that for these proteins UPF1 has a positive influence on their translation.

### uORFs as NMD features

The most prevalent NMD activating feature predicted for the doubly validated NMD targets was the presence of uORFs. Indeed, two of our confirmed cases that can be explained by uORFs have previously been identified as AS-NMD targets. *SFRS7 *(also known as 9G8) is already a well-characterised example of AS-NMD [55,62,76], our finding that some isoforms may also possess a uORF indicate that *SFRS7*'s post-transcriptional regulation may be more complex than previously appreciated, as recently described for *SFRS1 *(SF2/ASF) [66]. Furthermore, a *CCT8 *alternative isoform was reported to be sensitive to the translation inhibitor emetine by AS-sensitive microarray [32].

*A priori *it might be expected that all uORF containing mRNAs would be NMD sensitive. The termination codon of the uORF(s) would be premature with regard to the exon-exon junctions within the CDS, and would likely be at great distance from signals in the 3' UTR determining proper translation termination. This, however, does not appear to be the case as uORFs often mediate translational repression of the protein coding ORF without an accompanying decrease in mRNA abundance [26,77]. Moreover, inhibition of NMD in *C. elegans *results in the upregulation of only some transcripts bearing uORFs [21]. So what characteristics make a uORF baring transcript NMD sensitive? uORF length has been shown to be important; short open reading frames or those that have been translated more quickly favour translation reinitiation downstream [11,78,79], possibly by the remaining association of translation inititiation factors with the ribosome [80,81]. Reinitiation then inhibits NMD [11,78,79]. So NMD sensitive uORFs might be expected to be longer than average, or composed of sequence that results in slow translation. Ramani *et al*. (2009) found a weak correlation between the Kozak consensus at the start codon of the protein coding ORF and the extent to which the uORF containing transcript was up-regulated in NMD deficient *C. elegans *[21]. Calvo and colleagues examined the effect of various uORF characteristics on reduction in protein expression. They found that uORF containing 5' UTRs, when examined in a heterologous system, generally exerted a greater effect on protein abundance (average 58% decrease) than mRNA abundance (average 5% decrease). Similar trends were observed in published datasets they examined. The decreasing protein expression correlated with stronger context at the uAUG, a greater cap-uORF distance (both in the case of a single uORF) and, to a lesser extent, an increasing number of uORFs. In the case of a single uORF, uORF length and uORF-CDS distance did not correlate with the extent of protein repression. The absolute amplitude of changes in mRNA abundance are similar to those observed for our predicted uORF carrying genes. Unfortunately the relationship of these variables with decreasing mRNA abundance, which we might presume to be due to NMD, was not examined [82].

We have been unable to find any characteristics that distinguish the uORFs in our doubly validated NMD targets from others (data not shown). However, uORF mediated regulation is often complex, involving not only the uORFs themselves but also interactions with other conserved sequences and *trans*-acting factors [26,83,84]. Indeed, the relatively small fold changes in mRNA level observed during validation indicate that in each case only a proportion of the mRNA is NMD-sensitive, suggesting that some sort of probabilistic event is responsible. Thus, it is not clear that identification of a uORF is currently a powerful predictor of NMD sensitivity. A larger dataset and broader scope of analysis may prove more fruitful in finding some association between specific uORF characteristics, or groups thereof, and NMD-sensitivity.

### Autoregulation of HNRNPA2B1 via AS-NMD

We have identified a highly conserved example of autoregulatory AS-NMD within the *HNRNPA2B1 *gene. The hnRNP family of proteins plays many roles in RNA metabolism [85,86]. HNRNPA2B1 itself has been shown to regulate both alternative splicing [87] and mRNA stability [88]. Our data is consistent with HNRNPA2B1 activating splicing within it's own UTR to produce NMD sensitive forms that account for the decrease in total gene expression: over-expression of the HNRNPA2 isoform resulted in down-regulation of A2 and B1 isoform expression and the up-regulation of one of the NMD-sensitive exon junctions within the 3' UTR (Figure 6). For simplicity we measured two UTR exon-exon junctions that would always be expected to be NMD (in)sensitive, but the splicing patterns involved are almost certainly more complex.

While hnRNP A/B proteins were initially characterised as splicing repressors [86,89,90], it has recently been shown that intronic binding of hnRNP A1/A2 proteins can activate splicing, particularly of elongated introns [87,91]. This is thought to proceed by homophilic interactions between A2/A1 proteins bound at separate sites (or indeed heterophilic interactions between A1/A2 proteins and hnRNP F/H proteins) causing the looping out of portions of intron, which in turn results in promoting the splicing of said intron [87,92]. We have not examined whether HNRNPA2B1 directly binds to its own UTR, but motifs thought to represent bindings sites for HNRNPA1 and HNRNPA2B1 (taken from [91]) are clearly present in the HNRNPA2B1 3' UTR introns we identified (Additional file 3). This also raises the possibility that HNRNPA1 may also regulate these splicing events. Indeed increased expression of HNRNPA1 has been observed to correlate with decreased HNRNPA2/B1 in some cancer cell lines [93].

AS-NMD within hnRNP and SR protein genes has been shown to mediate quantitative regulation by repressing protein expression at inappropriate times [63,94-96] or providing homeostatic regulation of protein levels through autoregulatory negative feedback [32,36,58,60-66]. Our data is consistent with the later case. However, these two modes of regulation are not mutually exclusive, and it will be interesting to see whether biological circumstances can be identified where this AS-NMD event is used to repress expression of HNRNPA2B1. Indeed, up-regulation of HNRNPA2B1 levels has pathological associations. Increased HNRNPA2 expression has also been observed in pancreas and breast cancer [97,98], and in the foetal brain of Down's syndrome patients [99]. Underscoring the functional relevance of these increases, increased expression of HNRNPA2B1 and PTBP1 has been shown to be responsible for the predominance of the PKM2 isoform that is the hallmark of many types of cancer, promoting the aerobic glycolysis that is important for cell growth [100,101]. Furthermore, HNRNPA2 was recently shown to be responsible for splicing events that promote invasive migration of cancer cells in three-dimensional matrices [102]

## Conclusions

Despite the large number of changes in protein expression upon UPF1 knockdown, our two-stage validation shows that a relatively small fraction of them can be directly attributed to the action of NMD on the corresponding mRNA. This indicates that the role of NMD in directly regulating gene expression may be less prominent than previously suggested. The majority of the doubly-validated mRNAs contain computationally predicted uORFs, confirming this feature as an indicator of NMD sensitivity. We have also identified three examples of AS-NMD, including a highly conserved AS-NMD event that appears to mediate autoregulation of *HNRNPA2B1 *expression levels. This extends the observation that many RNA binding proteins auto-regulate their own expression through highly conserved elements. Consideration of this autoregulation will be important when examining biological situations, such as several types of cancer, where HNRNPA2B1 levels are deregulated.

## Methods

### Cloning

*Construction of pEGFPint*: an efficient artificial intron with associated exonic sequence was amplified from plasmid pY7 [35] using primers PY7INTF (TCTCAGCAAAGCGGCCGCTGCTGCGGGC) and PY7INTR2 (CTCTAGAGTCCAATTGCCTGCAGGCA) and *pfu *high fidelity polymerase. The pY7 intron is based on a β-globin intron while the exons are exons 2 and 3 from α-tropomyosin [35]. The resulting PCR product was cloned into pGEM T-easy (Invitrogen) and its identity confirmed by sequencing with T7 and SP6 primers. pEGFP-N1 (Promega) contains unique NotI and MfeI sites between the GFP stop codon and the SV40 polyA signals. The insert was liberated from pGEM T-easy by sequential digestion of the NotI and MfeI sites within PY7INTF and PY7INTR2 respectively and ligated with the corresponding fragment of pEGFP-N1. *Cloning of FLAG-hnRNPA2*: hnRNPA2 sequence was amplified from HeLa cell cDNA using primers hnRNPA2_F TACAGAATTCATGGAGAGAGAAAAGGAAC and hnRNPA2B1_R TCAGGTCGACGTATCGGCTCCTCCCACC. PCR was performed using 1.25 U Stratagene Native Pfu DNA Polymerase, 200 uM dNTPs and 400 uM primers. Cycling parameters: 95°C 2 min, [95°C 30 sec, 48°C 30 sec, 72°C 2 min]_35_, 72°C 5 min. The resulting PCR product was digested with EcoRI and SalI and ligated into pCI-NLS-FLAG [103], which allows expression of hnRNPA2 protein with N-terminal Flag tag and NLS, and 13 amino acid C-terminal tag. This cloning was confirmed by sequencing with T7 primer.

### Cell culture and transfections

HeLa cells were cultured under standard conditions in DMEM medium with glutamax (Invitrogen) and 10% fetal bovine serum. HeLa cells stably expressing pGFPint were generated by lipofectAMINE (Invitrogen) transfection of approximately 6 μg of pGFPint linearised at the *Apa LI *site. Transformant cell lines were then selected through growth in medium supplemented with 1 mg/mL G418 (Sigma) and isolated through ring cloning. Cell lines were then constantly maintained in growth medium containing G418 with the exception of when they were being used in an experiment. siRNA transfection of HeLa cells was performed using lipofectAMINE 2000 (Invitrogen) according to the "two-hit" protocol previously described [58]. The 19-mer sense target sequence and associated details of each siRNA are: control C2, 5'-GGUCCGGCUCCCCCAAAUG-3', 120 pmol/transfection or pGFPint experiments, 2.5 pmol/transfection 2D-DiGE expreiments [104]; Upf1_A, 5'-GAUGCAGUUCCGCUCCAUU-3', 120 pmol/transfection or pGFPint experiments, 2.5 pmol/transfection 2D-DiGE expreiments [105]; Upf1_B, 5'-GCUCCUACCUGGUGCAGUA-3', 2.5 pmol/transfection; Upf2, 5'-GGCUUUUGUCCCAGCCAUC-3', 120 pmol/transfection; SMG1_A, 5'-GUGAAGAUGUUCCCUAUGA-3', 120 pmol/transfection, Dharmacon siGENOME duplex D-005033-01-0050. Unless otherwise noted siRNA were designed and purchased from Dharmacon Inc. Samples were harvested for analysis 48 hours after the second transfection. In each case prior analysis had confirmed a high degree of knockdown at this time-point. This timepoint also represented a suitable trade-off between the need to allow sufficient accumulation of proteomic alterations directly resulting from UPF1 knockdown, while minimizing secondary effects. Such secondary effects might include false positives, which could arise if primary targets included, for example, transcription or translation factors, as well as false negatives due to compensatory mechanisms. Inhibition of NMD by cycloheximide was achieved by dosing HeLa cells to a final concentration of 10 ug/mL, or with an equivalent volume of DMSO, for 8 hours, as described [55].

### Analysis of protein expression by western blot

Extracts of total protein were obtained from tissue culture cells using RIPA buffer or ASB14 buffer. The concentration of these extracts was determined by Bradford assay [106]. Protein extracts were separated on 15% SDS-PAGE gels, transferred to PVDF membrane and then detected by a standard immunoblotting procedure followed by enhanced chemiluminescence detection. Primary antibodies used: rabbit anti-UPF1 [105], goat anti-UPF2 (Santa Cruz Biotechnology inc.), rabbit anti-GFP (Molecular Probes), rabbit anti-actin (Sigma), rabbit anti-ERK1 (invitrogen) Primary antibodies were detected by donkey anti-rabbit and donkey anti-goat antibodies conjugated to horseradish peroxidase.

### Analysis of mRNA expression by RT-PCR and QPCR

Total cellular RNA was harvested using TRI reagent (Sigma) according to the manufacturers instructions. 1 μg total RNA was treated with DNase I (Ambion) before oligo-dT reverse transcription using 200 U Superscript II (Invitrogen). PCR for the AS-NMD event within U2AF^35 ^was performed on 1/20^th ^of the RT reaction using the primers U2AF35_F: 5'-GCACAATAAACCGACGTTTAGCCAG-3', and U2AF35_R: 5'-TGGATCGGCTGTCCATTAAACCAAC-3' for 30 cycles with an annealing temperature of 59°C. AS-NMD events within the *TH1L *gene were examined using primers: Hs.517148_1_F 5'-GGGAGGAGGTGGATGACTTC-3' and Hs.517148_1_R 5'-GGTCAGCTTGGAAAGGAGTT-3' (intron retention, Figure 4B) for 38 cycles at 60°C annealing; Hs.517148_2_F 5'-ACTGCTGGACAGGATGGTTC-3' and Hs.517148_2_R 5'-TACCTGCGATGCTGTCATTC-3' for 40 cycles at 60°C annealing (alternative 5' splice site, Figure 4C). Electronic images of gels were captured using a MultiDoc-It Imaging System (UVP) and band intensities were analysed using the associated Doc-It(r) LS Image Analysis Software (UVP). Quantitative PCR (QPCR) was performed on a Rotor-GeneTM 6000 (QIAGEN) using a SYBR green master mix (Applied Biosystems). QPCR data was analysed using the comparative concentration module of the Rotor-Gene software, which is based on the second derivative maximum method described by Tichopad *et al. *[107]. Signal for the gene of interest (GOI) was normalized to *GAPDH *or *HPRT *levels then fold change in mRNA levels was calculated relative to the control sample. Gene specific QPCR primers were generally obtained from Primerbank [108] or designed using Primer3 plus [109], for sequences see Additional file 4. For each primer pair the formation of a single product was confirmed by melt curve analysis [110].

### Proteomics 2D-DiGE multi-gel study

Proteomics work was performed at the Cambridge Centre for Proteomics, Cambridge Systems Biology Centre, University of Cambridge. Extracts of total protein for analysis by 2D-DiGE were obtained from tissue culture cells using ASB14 lysis buffer and their concentrations quantified by DC Bradford protein assay (Biorad). For each sample 100 μg total protein was used for analysis. Protein separation was performed using a pI range of pH3-10 (non-linear strip) and a 12.5% SDS-PAGE gel. CyDye labeling, 2D protein separation, gel imaging, and analysis were performed as described previously using systems and software primarily obtained from GE healthcare [46,49,63,111]. PCA was performed using the software package SIMCA (Umetrics). Spots present in < 75% of the experimental samples (1061) were excluded, leaving 2021 for analysis. The first eight principal components (PCs) describing the data were calculated and the first two, PC_1 _and PC_2_, identified as significant. PC_1 _and PC_2 _result in a model with R^2 ^= 0.55 (goodness of fit) and Q^2 ^= 0.34 (goodness of prediction). Protein spots were excised both manually from gels stained with colloidal Coomassie, and automatically from fluorescently labelled gels using a CyProt-Picker robotics system (GE Healthcare). The protein constituents of the spots were then identified by LC-MS/MS sequencing of the tryptic peptides produced by in-gel digestion of the spots with trypsin. MS/MS fragmentation data were used to search the NCBI primary sequence database using MASCOT search engine [47].

### Bioinformatics

Computational prediction of AS-NMD was performed using a computational pipeline based on that described previously [112,113]. To begin with the Unigene cluster(s) [53] corresponding to each gene of interest was aligned to the genome sequence using SPA [54]. The resulting clusters of alignments were then processed by PASA [52]. PASA acts to subsume equivalent alignments to form a number of maximal transcript assemblies (termed maximum transcripts) that represent alternative mRNA isoforms consistent with the data from the alignment cluster. For each maximum transcript the largest ORF was defined and tested to determine whether the stop codon lay greater than 50 nucleotides upstream of an exon-exon junction. If this was the case for one or more of the maximum transcripts corresponding to a particular gene, then the gene was designated as possessing AS-NMD.

uORF prediction was performed on all the RefSeq transcripts and Ensembl annotated 5' UTRs corresponding to the genes of interest. In the case of the Refseq transcripts the largest ORF was defined and then the region directly upstream of this taken to be the 5' UTR. Then, for both sets of UTRs, ORFs beginning with ATG were sought for in the forward three reading frames.

Finally, potential NMD sensitive maximum transcripts were then scored according to the number of peptides identified by mass spectrometry that were present within the protein sequence encoded by the largest ORF of the maximum transcript. Only transcripts encoding all of the identified peptides were considered as being potentially responsible for the observed upregulation of protein spots.

### 3' Race

1 μg of DNAase I treated total RNA from HeLa cells having undergone UPF1 knockdown was subject to reverse transcription by superscript II (Invitrogen) using an oligo-dT primer with the 5' adaptor sequence: 5'-GGACGCGTAAGCTTGTCGAC-3'. PCR was then performed using a primer with the adaptor sequence and primers within both the terminal coding exon of hnRNPA2/B1 (A2B15'1: TTTGGTGGTAGCAGGAACAT, A2B15'2: TGGAGGAAACTATGGTCCAG) and within predicted portions of the 3' UTR (A2B15'3: TTGGTTCCTTCAGTGGTGTT, A2B15'4: TGCTGCCACAAAGACTGTAA). Sequences from these reactions were cloned into pGEM T-easy (Invitrogen) and sequenced.

### Sorting of cells by flow cytometry

2 × 10^5 ^HeLa cells/well of a 6 well plate were co-transfected with 1 μg pCI-NLS-FLAG hnRNPA2 (or the empty pCI-NLS-FLAG vector) and an equivalent molar amount of pEGFP-N1 (927.94 ng) using Lipofectamine 2000 (Invitrogen). 48 hours later cells were harvested for flow cytometry by trypsination. Cells from four wells were pooled for each replicate and resuspended in 1 mL DMEM + 2% FCS. For each replicate 1 × 10^6 ^GFP positive cells were collected using a MoFlo high-speed cell sorter (Beckman Coulter). GFP flourescence was detected using a 530/30 filter and live/dead cells discriminated with To-Pro-3 staining, detected using a 670/30 filter. Cells were gated based on forward and side scatter to eliminate debris and then doublet discrimination was carried out to ensure only single cells were sorted (Additional file 5).

## Authors' contributions

NJM, KL & CWJS designed the study, NJM performed the experiments and analysed the data, LYT performed experiments pertaining to HNRNPA2B1 autoregulation, NP & MZ performed bioinformatic prediction of AS and NMD activating features, KL assisted with proteomics data analysis, NJM & CWJS wrote the paper. All authors read and approved the final manuscript.

## Supplementary Material

Additional file 1 **Protein spots of interest excised for identification by mass spectrometry**. Cy2 image of one gel from the 2D-DiGE multi-gel study. The 85 up-regulated spots excised for identification by mass spectrometry are circled in red and the 17 down-regulated spots excised are circled in blue. Each spot is labelled with its number from Additional file 2. Click here for file

Additional file 2 **XLS file containing the collated data from the 2D-DiGE multi-gel study, peptide sequence data and protein IDs produced by mass spectrometry, computational prediction of NMD activating features and QPCR based validation of changes in mRNA expression**. Click here for file

Additional file 3 **A. Novel 3' UTR sequence of HNRNPA2B1 constructed from RACE tags**. Exon sequence is capitalised. HNRNPA1 and A2/B1 motifs from [91] are emboldened and underlined. The small UTR intron present in the Refseq UTR is outlined in black. **B**. Sequences of the 3' RACE tags illustrated in Figure 6. Click here for file

Additional file 4 **List of all the QPCR primer used in this study, including sequence and design source**. Click here for file

Additional file 5 **Sorting of GFP positive cells by flow cytometry**. Representative plots of the cell sorting used in Figure 6. Cell events are denoted by dots. Increasingly "hot" colours represent increasing density of cell events. In each panel, the cells selected for further sorting are outlined by a polygon (termed a gate), and the inlaid number indicates the percentage of cells at that stage within the gate. **A**. Cells were gated based on forward and side scatter to eliminate debris. **B**. Doublet discrimination was carried out to ensure only single cells were sorted. **C**. Live/dead cells were discriminated with To-Pro-3 staining, detected using a 670/30 filter. **D**. GFP fluorescence was detected using a 530/30 filter. Click here for file
