# Supplementary material for: Expression proteomics of UPF1 knockdown in HeLa cells reveals autoregulation of hnRNP A2/B1 mediated by alternative splicing resulting in nonsense-mediated mRNA decay
Source: BMC Genomics. 2010 Oct 14;11:565. doi: 10.1186/1471-2164-11-565 (PMC3091714; doi:10.1186/1471-2164-11-565)
Supplement: Additional file 3 — A. Novel 3' UTR sequence of HNRNPA2B1 constructed from RACE tags. Exon sequence is capitalised. HNRNPA1 and A2/B1 motifs from [91] are emboldened and underlined. The small UTR intron present in the Refseq UTR is outlined in black. B. Sequences of the 3' RACE tags illustrated in Figure 6. [file 1471-2164-11-565-S3.doc]

**A**

GAAACTATGGTCCAGGAGGCAGTGGAGGAAGTGG**GGGT**TATGGTGGGAGGAGCCGATACTGAGCTTCTTCCTATTTGCCAT**GGgt**aagtagcttttgagttttacaattattattatcttgggagacatagctgcaggagtaaaagctttt**tagg**atcatgttatctttccttaaaatctggttagatggataatttcataacctattttttttttactctttacttctgttgaaacaggcttcactgtataaa**tagg**agaggatgagagcccagaggtaacagaacagcttcaggttatcgaaataacaatgttaaggaaactcttatctcagtcatgcataaatatgcagtgatatggcagaagacaccagagcagatgcagagagccattttgtgaatggattggattatttaataacattaccttactgtggaggaaggattgtaaaaaaaaatgcctttgagacagtttcttagctttttaattgttgtttctttctagtggtctttgtaagagtgtagaagcattccttctttgataatgttaaatttgtaagtttcaggtgacatgtgaaaccttttttaagatttttctcaaagttttgaaaagctattagccaggatcatggtgtaataagacataacgtttttcctttaaaaaaatttaagtgcgtgtg**tagag**ttaagaagctgttgtacatttatgatttaataaaataattctaaaggaaattgtgtaattatagactttttattttaaataagttaaggagt**gggt**agtataattaaggtccgttgcaaagctgttgttatatttgtataagataaatgctggtcagatgtaagtgtgttgtctgcaattcatcaggattaaattatgtagataacttaagggatatctctgcaaggagaaacacctttttagATCTTTTAGATGCTGCTTCTTCAATGCAAGGAAAGGAAATAACCCCAGCGAGGTACTCTTCAGGGACACAGGTCTAGTACAAGAGAACTCTTGACGGCTACTAAGTTCAGCCAGTCTTAAAAAACTGTGCTGTTTCTACAAAACTTTAACTACAGTAGTTTATAAGGATGCCAACGAAAGCTGA**GGGT**G**TAGAG**CAAAATAGTTCTAAGCTTCAGTTAAACTTCTT**TAGg**taagatcttatttacttttcctttcttaattttcctccctaaaagataaactaatactcttaaatggtctttcagtatagtggttcttacgtagtttaacatagctataaattgagtttaacaatttataaactcaagagaataatttttataaaccctgttttccaatctgtcatttacttaaattattttggttgtttttccctttttttccttcttttcccaccccctccccctccatgtgaagattt**gggt**gcttaacatatcatttttttccctgccggaattttagCATTGATATGAACCATGGACAAGTATATTCTGCTGCCACAAAGACTGTAAAGTGCTTCATTTCAACAGCTGAGGCAAGCCAAGTGATCATTAATAAAGCTTTTCTTGGTTCCTTCAGTGGTGTTGGTAGTAAAATGg**tagg**taaaagt**tagg**ctgcaagttcaataaatcatgagatttcccatcgttacacccttgtgtattcacatttcttggatcaaacattttgagtgaac***tagg*ggt**ttttattaaagacatttgttgtatttatggttgtaactgtacatgcttatcaggatgagactgaaagaagg**taggg**caaaaatggttgaatctattttcagatagtagttcatacttgagtgaagtgtcttgtctgcattatgaagcctggtatgtatccagtactaaa**tagg**t**gggt**taaatgtggtaattctagttcagtgtcttaccctgaagagaaagttg**tagg**ttggctgttgaaattcattccttagatatgatcagtttgattgcccggctttattgcctttacaggaatgtgatactcagggcttactctatacaccaatgagtcttctttgatcctaagaccaccactgaagttgtt**tagg**ttcttttggacaaacatgataaacttcttcagatactttttttttcctttggcagGAAGGTGTCTTGCTGCAGGTAACTAATGAAGAAGTGGTCAACCACAGAGTCTTCAAGAAATAAGAAATTCTGTACCATCTGAAAGTAGTTCTTGTTGGTGCCTT

Intron 1: 844

Intron 1a: 1336

Intron 2: 293

Intron 3: 532

**B**

>race_r2_lig1_8_30_639

GCGGGAATTCGATTTTTGGTGGTAGCAGGAACATGGGGGGACCATATGGTGGAGGAAACTATGGTCCAGGAGGCAGTGGAGGAAGTGGGGGTTATGGTGGGAGGAGCCGATACTGAGCTTCTTCCTATTTGCCATGGCATTGATATGAACCATGGACAAGTATATTCTGCTGCCACAAAGACTGTAAAGTGCTTCATTTCAACAGCTGAGGCAAGCCAAGTGATCATTAATAAAGCTTTTCTTGGTTCCTTCAGTGGTGTTGGTAGTAAAATGGAAGGTGTCTTGCTGCAGGTAACTAATGAAGAAGTGGTCAACCACAGAGTCTTCAAGAAATAAGAAATTCTGTACCATCTGAAAGTAGTTCTTGTTGGTGCCTTCATTTAAAAAGCACTCTTTAAAATAAAAGGGAAATGTTTTCTGATAAAACAAACATTTAGTTGAGGTTCTTGATATAAAACAATTACAAAATGAGTGTTGTTTGTAAAACAGTAACATCAAATTGGCTAGAGAGATAAATGTATCATGTTTTAAATTAGGTTTTGTGAGTAGACAGATTACAATTCTATTTTAAATATAAAGTTTATAAAATAAATACTTTTTGTATCC

//

>race_r3_lig1_9_30_630

ATTCGATTTTTGGTGGTAGCAGGAACATGGGGGGACCATATGGTGGAGGAAACTATGGTCCAGGAGGCAGTGGAGGAAGTGGGGGTTATGGTGGGAGGAGCCGATACTGAGCTTCTTCCTATTTGCCATGGATCTTTTAGATGCTGCTTCTTCAATGCAAGGAAAGGAAATAACCCCAGCGAGGTACTCTTCAGGGACACAGGTCTAGTACAAGAGAACTCTTGACGGCTACTAAGTTCAGCCAGTCTTAAAAAACTGTGCTGTTTCTACAAAACTTTAACTACAGTAGTTTATAAGGATGCCAACGAAAGCTGAGGGTGTAGAGCAAAATAGTTCTAAGCTTCAGTTAAACTTCTTTAGCATTGATATGAACCATGGACAAGTATATTCTGCTGCCACAAAGACTGTAAAGTGCTTCATTTCAACAGCTGAGGCAAGCCAAGTGATCATTAATAAAGCTTTTCTTGGTTCCTTCAGTGGTGTTGGTAGTAAAATGGAAGGTGTCTTGCTGCAGGTAACTAATGAAGAAGTGGTCAACCACAGAGTCTTCAAGAAATAAGAAATTCTGTACCATCTGAAAGTAGTTCTTGTTGGTGCCTT

//

>race_r4_lig1_10_30_687

GCGGGAAATTCGATTTTTGGTGGTAGCAGGAACATGGGGGGACCATATGGTGGAGGAAACTATGGTCCAGGAGGCAGTGGAGGAAGTGGGGGTTATGGTGGGAGGAGCCGATACTGAGCTTCTTCCTATTTGCCATGGCATTGATATGAACCATGGACAAGTATATTCTGCTGCCACAAAGACTGTAAAGTGCTTCATTTCAACAGCTGAGGCAAGCCAAGTGATCATTAATAAAGCTTTTCTTGGTTCCTTCAGTGGTGTTGGTAGTAAAATGGAAGGTGTCTTGCTGCAGGTAACTAATGAAGAAGTGGTCAACCACAGAGTCTTCAAGAAATAAGAAATTCTGTACCATCTGAAAGTAGTTCTTGTTGGTGCCTTCATTTAAAAAGCACTCTTTAAAATAAAAGGGAAATGTTTTCTGATAAAACAAAAAAAAAAAAAAAAGTCGACAAGCTTACGCGTCCAATCACTAGTGAATTCGCGGCCGCCTGCAGGTCGACCATATGGGAGAGCTCCCAACGCGTTGGATGCATAGCTTGAGTATTCTATAGTGTCACCTAAATAGCTTGGCGTAATCATGGTCATAGCTGTTTCCTGTGTGAAATTGTTATCCGCTCACAATTCCACACAACATACGAGCCGGAAGCATAAAGTGTA

//

>race_r6_lig2_2_30_700

GGGAATTCGATTTGGAGGAAACTATGGTCCAGGAGGCAGTGGAGGAAGTGGGGGTTATGGTGGGAGGAGCCGATACTGAGCTTCTTCCTATTTGCCATGGCATTGATATGAACCATGGACAAGTATATTCTGCTGCCACAAAGACTGTAAAGTGCTTCATTTCAACAGCTGAGGCAAGCCAAGTGATCATTAATAAAGCTTTTCTTGGTTCCTTCAGTGGTGTTGGTAGTAAAATGGAAGGTGTCTTGCTGCAGGTAACTAATGAAGAAGTGGTCAACCACAGAGTCTTCAAGAAATAAGAAATTCTGTACCATCTGAAAGTAGTTCTTGTTGGTGCCTTCATTTAAAAAGCACTCTTTAAAATAAAAGGGAAATGTTTTCTGATAAAACAAACATTTAGTTGAGGTTCTTGATATAAAACAATTACAAAATGAGTGTTGTTTGTAAAACAGTAACATCAAATTGGCTAGAGAGATAAATGTATCATGTTTTAAATTAGGTTTTGTGAGTAGACAGATTACAATTCTATTTTAAATATAAAGTTTATAAAATAAATACTTTTTGTATCCAAAAAAAAAAAAAAAAGTCGACAAGCTTACGCGTCCAATCACTAGTGAATTCGCGGCCGCCTGCAGGTCGACCATATGGGAGAGCTCCCAACGCGTTGGAT

//

>race_r9_lig2_9_25_700

CGGCCGCGGGAATTCGATTTGGAGGAAACTATGGTCCAGGAGGCAGTGGAGGAAGTGGGGGTTATGGTGGGAGGAGCCGATACTGAGCTTCTTCCTATTTGCCATGGCATTGATATGAACCATGGACAGGTATATTCTGCTGCCACAAAGACTGTAAAGTGCTTCATTTCAACAGCTGAGGCAAGCCAAGTGATCATTAATAAAGCTTTTCTTGGTTCCTTCAGTGGTGTTGGCAGTAAAATGGAAGGTGTCTTGCTGCAGGTAACTAATGAAGAAGTGGTCAACCACAGAGTCTTCAAGAAATAAGAAATTCTGTACCATCTGAAAGTAGTTCTTGTTGGTGCCTTCATTTAAAAAGCACTCTTTAAAATAAAAGGGAAATGTTTTCTGATAAAAAAAAAAAAAAAAAAAAAAAGTCGACAAGCTTACGCGTTAATCACTAGTGAATTCGCGGCCGCCTGCAGGTCGACCATATGGGAGAGCTCCCAACGCGTTGGATGCATAGCTTGAGTATTCTATAGTGTCACCTAAATAGCTTGGCGTAATCATGGTCATAGCTGTTTCCTGTGTGAAATTGTTATCCGCTCACAATTCCACACAACATACGAGCCGGAAGCATAAAGTGTAAAGCCTGGGGTGCCTAATGAGTGAGCTAACTCACATTAATTGCGTTGCG

//

>race_r11_lig3_3_25_647

CGGCCGCGGGAATTCGATTAGTATTTATTTTATAAACTTTATATTTAAAATAGAATTGTAATCTGTCTACTCACAAAACCTAATTTAAAACATGATACATTTATCTCTCTAGCCAATTTGATGTTACTGTTTTACAAACAACACTCATTTTGTAATTGTTTTATATCAAGAACCTCAACTAAGTGTTTGTTTTATCAGAAAACATTTCCCTTTTATTTTAAAGAGTGCTTTTTAAATGAAGGCACCAACAAGAACTACTTTCAGATGGTACAGAATTTCTTATTTCTTGAAGACTCTGTGGTTGACCACTTCTTCATTAGTTACCTGCAGCAAGACACCTTCCATTTTACTACCAACACCACTGAAGGAACCAAAATCACTAGTGAATTCGCGGCCGCCTGCAGGTCGACCATATGGGAGAGCTCCCAACGCGTTGGATGCATAGCTTGAGTATTCTATAGTGTCACCTAAATAGCTTGGCGTAATCATGGTCATAGCTGTTTCCTGTGTGAAATTGTTATCCGCTCACAATTCCACACAACATACGAGCCGGAAGCATAAAGTGTAAAGCCTGGGGTGCCTAATGAGTGAGCTAACTCACATTAATTGCGTTGCGCTCACTG

//

>race_r18_lig4_9_25_317

GGCCGCGGGAAATTCGATTTGCTGCCACAAAGACTGTAAAGTGCTTCATTTCAACAGCTGAGGCAAGCCAAGTGATCATTAATAAAGCTTTTCTTGGTTCCTTCAGTGGTGTTGGTAGTAAAATGGAAGGTGTCTTGCTGCAGGTAACTAATGAAGAAGTGGTCAACCACAGAGTCTTCAAGAAATAAGAAATTCTGTACCATCTGAAAGTAGTTCTTGTTGGTGCCTTCATTTAAAAAGCACTCTTTAAAATAAAAGGGAAATGTTTTCTGATAAAACAAAAAAAAAAAAAA

//
